# Predicting vitamin E and C consumption intentions and behaviors among factory workers based on protection motivation theory

**DOI:** 10.1186/s12199-018-0742-z

**Published:** 2018-10-24

**Authors:** Sahar Mohammad Nabizadeh, Parvaneh Taymoori, Mohammad Saleh Hazhir, Mehra Shirazi, Daem Roshani, Behzad Shahmoradi

**Affiliations:** 1 Environmental Health Research Center, Research Institute for Health Development, Kurdistan University of Medical Sciences, P.O. Box: 6618634683, Sanandaj, Iran; 2 Department of Nutrition, Faculty of Medicine, Kurdistan University of Medical Sciences, Sanandaj, Iran; 3 Women, Gender, and Sexuality Studies, School of Language, Culture and Society, Oregon State University, Corvallis, USA; 4 Social Determinants of Health Research Center, Kurdistan University of Medical Sciences, Sanandaj, Iran

**Keywords:** Protection motivation theory, Vitamin E, Vitamin C, Structural equation modeling

## Abstract

**Background:**

Study of antioxidant vitamin consumption behavior, especially in high-risk groups with high exposure to toxic metals to reduce metal toxicity, is emphasized. This study aims to examine the structural relationships between knowledge, protection motivation theory constructs, and vitamin E and C consumption behavior among cement factory workers.

**Methods:**

Protection motivation theory and food frequency questionnaires were completed by 420 factory workers. Data were subjected to structural equation modeling to examine associations between knowledge, protection motivation theory constructs, and vitamin E and C consumption behavior. Efficacy of current recommended models was also explored.

**Results:**

Structural equation modeling showed high explained variance within the constructs of protection motivation theory for vitamin E and C consumption behavior and intention (56–76%). The overall fit of the structural models was acceptable for both vitamin E and C behavior. Knowledge, self-efficacy, response efficacy, and perceived vulnerability predicted intention, which in turn predicted vitamin consumption behavior. Significant relationships between knowledge and self-efficacy, response efficacy, perceived vulnerability, and perceived severity were also found, while self-efficacy and response efficacy showed significant relationships with vitamin E and C consumption behavior.

**Conclusions:**

Considering that response efficacy, self-efficacy, and intention showed as strong predictors of vitamin E and C consumption behavior, specific attention should be paid to coping appraisals and intention when designing intervention plans. Additionally, establishing the predicting role of knowledge for protection motivation theory constructs and protective behaviors should be integrated into intervention programs.

## Introduction

Dusts of the cement industries, which include high levels of toxic metals and some toxic compounds, are harmful for human health [1]. Results of the previous studies that focused on the determination of the levels of metals in the blood of the cement factory workers revealed that workers and the residents of the neighboring communities were at the risk of metal poisoning to which they were exposed [2, 3]. Recent studies have shown that toxic metal exposure can lead to cardiovascular, renal, immune, gastrointestinal, and nervous system problems even at low concentrations [4, 5]. Studies have suggested the protective role of antioxidant vitamins such as vitamins E and C against toxic injury and other disease [5, 6]. Different reports showed that these vitamins can provide a convenient method of reducing metal poisoning, perhaps by increasing renal clearance and reducing the intestinal absorption of the metals [6, 7].

Micronutrient deficiency, particularly of vitamins, is one of the major problems of global health [8]. Previous studies in Iran have reported that individuals have not, as yet, adopted the minimum recommendations for the consumption of vitamins [9]. Such problems in Iran and elsewhere indicate that there is urgent need for health promotion programs to increase knowledge of antioxidant vitamins, particularly in high-risk groups as factory workers with high exposure to toxic metals.

**Fig. 1:**
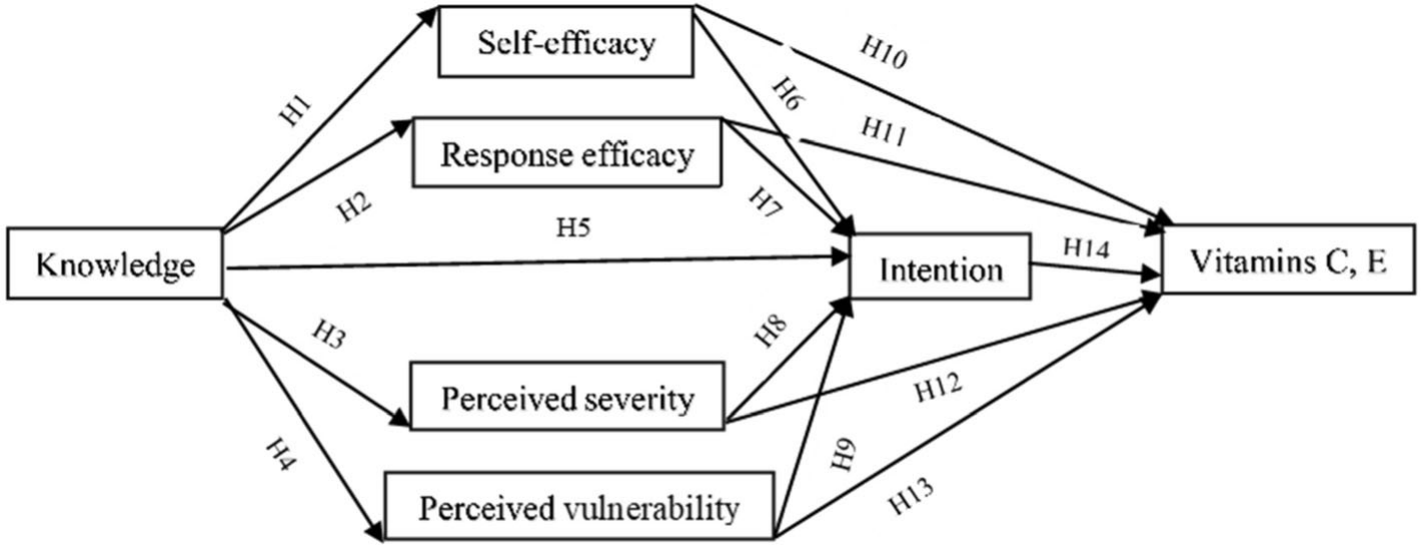
Path diagram of the hypothesized relationships. H, hypothesis

Protection motivation theory (PMT) has been applied to the prediction of a range of health protective behaviors, including exercise, cervical cancer screening, breast and testicle self-examination, smoking, and the consumption of health-enhancing foods [10, 11]. No studies have yet applied it to vitamin E and C consumption behavior in Iranian factory workers. PMT proposes that both individual and environmental factors can provide encouragement for engaging in protective behaviors and that the effects of such factors are mediated by individual cognitive processes [12]. The individual may consider the extent to which the potential threat poses harm [perceived severity] or their own risk factors for being susceptible to a given threat [perceived vulnerability] [13]. The individual first considers their perceptions of whether or not a given protective response will be able to prevent a given threat [response efficacy] and whether they will be able to employ the protective response in a way in which it will be effective in preventing the potential threat [self-efficacy] [14]. The summation of considerations associated with the threat and coping appraisal processes produces the individual’s “protection motivation (intention)” when the individual perceives a serious threat, believes in the efficacy and the capability of a potential protective response, and is likely to engage in the given protective response [15]. Rogers’s theoretical PMT model posited that linear relationships exist between the threat and coping appraisals, intention, and behavior [13]. In this study, these relationships and the hypothesized path diagram are shown in Fig. 1.

Previous studies suggest that coping appraisal variables provided the strongest behavior and intention predictions [13, 15]. Here, relationships between coping appraisal variables, intention, and vitamin consumption behavior are expressed in hypotheses H6, H7, H10, and H11. Also, according to previous PMT research, intention had a significant role and strong predictor on different behaviors [16, 17]. The direct relationship of intention to vitamin consumption behavior is expressed in H14. Moreover, relationships between threat appraisal variables, intention, and vitamin consumption behavior are expressed in hypotheses H8, H9, H12, and H13. Theoretical PMT model indicated a relationship between the threat appraisal and intention of behavior and found that increases in perceived severity or vulnerability lead to higher protection behavior performance [13]. Additionally, the results suggested that knowledge plays an important role on PMT constructs [18, 19]. The relationships between knowledge, coping appraisal, threat appraisal, and intention are expressed in H1 to H5.

While previous studies examined PMT constructs on different behaviors (exercise, cervical cancer screening, breast and testicle self-examination, alcohol consumption, etc.) in different populations and other researches applied structural equation modeling (SEM) method to examine relations between the PMT constructs, in our knowledge, this is the first study addressing the structural relationships of PMT constructs and knowledge on consumption behavior of vitamins E and C in cement factory workers.

## Methods

Four hundred twenty participants were randomly assigned using a random number table from a possible 500 male cement factory workers [20]. All workers provided written, informed consent, and participation was voluntary.

### Measures

PMT constructs were adopted based on previous food consumption behavior studies [12, 17]. To obtain content validity, five experts (Ph.D. level) in health behavior, psychology, nutrition, and instrument design evaluated the researchers Farsi language questionnaire. To ensure the clarity of the questionnaire, a pilot examination was performed with 30 workers. The questionnaire was then modified based on the feedback received. Demographic characteristics, knowledge, and PMT constructs were assessed by a self-reported questionnaire.

Perceived severity was measured with 12 items, e.g., “Heavy costs of the treatment of the probable cancer made me and my family feel anxious.” The 2-week test/retest reliability of the perceived severity scale was *r* = 0.89, and the alpha coefficient (*α*) was 0.83. Perceived vulnerability was measured with 7 items, e.g., “Because you do not consume vitamins regularly, your cardiovascular system has already begun deteriorating.” The 2-week test/retest reliability of the perceived vulnerability scale was *r* = 0.71, and the (*α*) was 0.78. Self-efficacy was measured by 8 items, e.g., “I believe that I have the ability to successfully use antioxidant vitamins.” The 2-week test/retest reliability of the self-efficacy scale was *r* = 0.77, and the (*α*) was 0.90. Response efficacy was measured with 8 items, e.g., “fruit and vegetable consumption will ensure that I avoid the health problems associated with vitamins deficiency.” The 2-week test/retest reliability of the response efficacy was *r* = 0.78, and the (*α*) was 0.83. Intention to consume vitamins E and C was measured with one item asking participants about their intention during the next months. The 2-week test/retest reliability of the intention scale was *r* = 0.69, and the (*α*) was 0.88. Participants’ knowledge was assessed by 13 items, which measured workers’ capabilities to understand information about risk factors and antioxidant vitamins (*α* = 0.83). Possible responses were correct answers = 1 and incorrect or do not know answers = 0. Perceived severity, perceived vulnerability, response efficacy, self-efficacy, and intention were assessed using a 5-point Likert scale (from 1 = strongly disagree to 5 = strongly agree).

### Vitamin E and C consumption

There are several methods used for dietary data evaluation, with varying validity depending on the detail required [21]. The most effective and commonly used tool to collect data at the different population level is the food frequency questionnaire (FFQ) [22]. As FFQ is cost-effective, poses less burden on participants, and can be analyzed rapidly as compared to other dietary assessment techniques, it is now the most commonly used standard dietary tool in nutritional studies all over the world [23, 24]. In this study, the vitamin E and C intake were obtained using a FFQ, which contained questions regarding a list of foods, frequency of intake, and a standard serving size for each commonly consumed by Iranians [22, 25]. In the analyses of validity, correlation coefficient was mean *r* = 0.53, and in reproducibility, correlation coefficient was mean *r* = 0.59. Whereas Iranian food composition table (FCT) is not comprehensive and complete, for some food items, the United States Department of Agriculture Food Consumption Table (USDA) FCT [25] was used, too. Frequencies were formatted to recall food consumption during the previous year on a daily, weekly, or monthly basis. Foods were classified into the following categories: red meats, organ meats, poultry, fish and other seafood, eggs, carbonated drinks, dairy products, fruits and dried fruits and juices, vegetables, legumes and nuts, potatoes, whole grains, refined grains, salty snacks and vegetables, animal fats, vegetable oils, olives, sugars, sweets and desserts, tea, and coffee. Portion sizes of consumed foods were converted to grams by using household measures [23, 25]. Daily vitamin E and C consumption for participants were computed by Nutritionist IV software which was designed for the evaluation of Iranian foods. Results were expressed as milligrams of vitamins E and C per day (mg/day).

### Data analysis

Collected data were analyzed using SPSS 24.0. SEM estimation was performed using the maximum likelihood function with AMOS 24.0. Values were given in means and standard deviations. The comparative fit index (CFI), goodness of fit index (GFI), Tucker Lewis index (TLI), root mean square error of approximation (RMSEA), the model chi-square (*χ*^2^), and $$ \raisebox{1ex}{${\chi}^2$}\!\left/ \!\raisebox{-1ex}{$\mathrm{df}$}\right. $$ were used to evaluate the overall model fitness. Acceptable model fit is detected when CFI, GFI, and TLI values are > 0.90, RMSEA is < 0.10 [14], and $$ \raisebox{1ex}{${\chi}^2$}\!\left/ \!\raisebox{-1ex}{$\mathrm{df}$}\right. $$ is < 2–5 [26]. Moreover, *R*^2^, the determination coefficient, was performed showing the variance percentage to establish the predictive power of the model.

## Results

The mean age of workers was 34.36 (standard deviation = 4.72) years; education status was 7.1% under diploma, 41.0% diploma, 34.8% bachelor science, and 17.1% master science; marital status was 72.4% married and 27.6% single; and the mean working experience of workers was 8.51 (standard deviation = 4.30) years. The scale means for each measured variable in the models are displayed in Table 1.

**Table 1:** Means and standard deviation of PMT constructs (*N* = 420)

Variables	Mean	Standard deviation
Knowledge	1.65	0.54
Self-efficacy	2.76	0.54
Response efficacy	3.33	0.64
Perceived severity	2.81	0.63
Perceived vulnerability	3.11	0.55
Intention	2.25	1.13
Vitamin E (mg/day)	1.57	1.17
Vitamin C (mg/day)	13.10	8.10

**Fig. 2:**
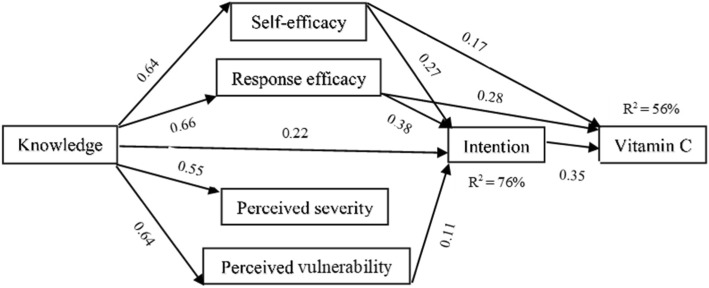
PMT path model showing the significant standardized beta coefficients of vitamin C behavior

**Fig. 3:**
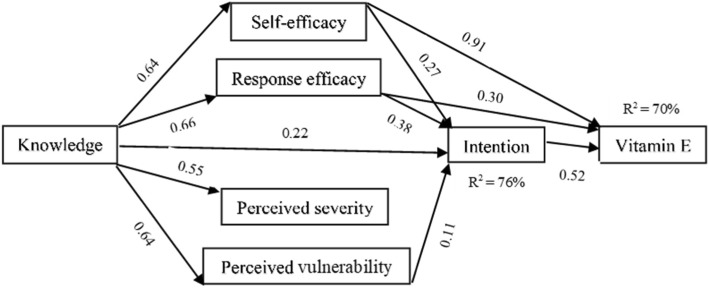
PMT path model showing the significant standardized beta coefficients of vitamin E behavior

The resulting model of vitamin E and C consumption behavior is reported in Figs. 2 and 3. These models show a good fit with the empirical data (Table 2). Several significant predictors were found and are expressed as standardized beta values.

**Table 2:** Goodness of fit indices for the vitamin E and C consumption behavior models testing PMT

	*χ* ^2^	Df	*p*	CFI	GFI	$$ \raisebox{1ex}{${\chi}^2$}\!\left/ \!\raisebox{-1ex}{$\mathrm{df}$}\right. $$	RMSEA	TLI
Vitamin C	5.04	4	0.28	0.99	0.99	1.26	0.02	0.99
Vitamin E	0.79	1	0.37	0.99	0.99	0.79	0.01	0.99

With regard to vitamin C (Fig. 2), the relationship between the constructs and vitamin consumption behavior is explained. The coefficient correlations showed significant relationships between behavior and self-efficacy, response efficacy, and intention (*p* < 0.0001–0.01), explaining 56% of the variance. These relationships confirmed hypotheses H10, H11, and H14. Additionally, the relationship between the constructs and intention is explained. There are significant relationships between intention and knowledge, self-efficacy, response efficacy, and perceived vulnerability (*p* < 0.0001–0.01), explaining 76% of the variance. Hypotheses H5, H6, H7, and H9 were confirmed by these findings. Finally, knowledge showed significant relationships with self-efficacy, response efficacy, perceived vulnerability, and perceived severity (*p* < 0.0001–0.01) as were predicted by hypotheses Hl to H4. Perceived severity was not significantly linked to intention and behavior, and perceived vulnerability was also not significantly linked to behavior.

When assessing vitamin E (Fig. 3), the relationship between the constructs and vitamin consumption behavior is explained. Here, self-efficacy had the greatest effect on behavior, followed by intention and response efficacy (*p* < 0.0001–0.01), explaining 70% of the variance. Hypotheses H10, H11, and H14 were confirmed by this. Further, the relationship between the constructs and intention is explained. The coefficient correlations indicated significant associations between intention and knowledge, self-efficacy, response efficacy, and perceived vulnerability (*p* < 0.0001–0.01), explaining 76% of the variance. The findings confirmed hypotheses H5, H6, H7, and H9. There were also significant relationships between knowledge and self-efficacy, response efficacy, perceived vulnerability, and perceived severity (*p* < 0.0001–0.01). Thus, hypotheses H1 to H4 were confirmed. Perceived vulnerability was not significantly linked to vitamin E consumption behavior, and perceived severity was also not significantly linked to intention and behavior.

## Discussion

This is the first study addressing the structural relationships of PMT constructs and knowledge on consumption behavior of vitamins E and C in cement factory workers. There were few or no earlier studies to refer to or rely upon to predict an outcome. This research was performed among a population of cement workers in a deprived area where there was not available information related to antioxidant vitamin benefits and toxic metals. Attention to research among workers who have high exposure to toxic metals and have low level of knowledge about antioxidant benefits is an area of study with the potential for high impact on the quality of life. In addition, to establish the relationships of the main components of PMT, knowledge was also used to predict intention and behavior, which has been ignored in many previous studies [27, 28], and the relationship of knowledge to other variables in our study indicates a potential intervention model using education on the value of antioxidant consumption. We were able to objectively measure behavior around vitamin consumption, and despite the different predictive power of constructs to predict vitamin E and C consumption, the model was structurally sound. As such, it could be appropriate to predicting the other antioxidant consumption behavior. This study adds to existing research that knowledge and PMT constructs, particularly copping appraisal, predicted intention, which in turn predicted vitamin consumption behavior. Understanding these relationships is important when designing dietary educational intervention plans.

Our results showed high explained variance within the constructs of PMT for the consumption of vitamins C (56%) and E (70%), and 76% for intention. Chin [29] recommended the amounts of 0.67, 0.33, and 0.19 for the variance percentage in a model as substantial, moderate, and weak, respectively. Our results were consistent with the results of Cox et al. in explaining a high percentage (59–69%) of the variation of intention to consume functional foods and supplements in an applied PMT model among an Australian population [27]. While in this study, similar constructs predicted the behavior of both vitamin E and C consumption, their predictive power was different for each vitamin’s consumption behaviors. A comparison of the results of this study to previous findings indicates a difference in the predictive power of the model for related behaviors. The reason for this difference is likely related to different behaviors, and as such, additional studies are necessary to address the predictive power of the model for related behaviors.

We showed that knowledge about vitamins E and C predicted an increase in self-efficacy and response efficacy and subsequently might have influenced intention. Furthermore, knowledge had a direct effect on perceived severity, perceived vulnerability, and intention. Studies have shown that knowledge plays an important role on the recommended behavioral changes [30, 31]. Although vitamin deficiency has been reported in previous studies [9], attention to research among workers who are exposed to toxic metals and their knowledge of the role of antioxidants has been ignored and more attention should be paid. More importantly, because of the high impact of knowledge on PMT constructs showed in the present research, further studies are indicated to examine relationships between knowledge and PMT constructs for different behaviors and population, as well as studying educational interventions to understand how changes in knowledge levels can influence constructs of PMT and related behaviors.

The results revealed self-efficacy and response efficacy (coping appraisal) were significantly linked with vitamin consumption behavior and intention, believing themselves able to successfully perform the recommended behavior appears to have been a prerequisite for intending to adopt that behavior [32]. Results of path analysis constructs of PMT among university students indicated significant relationship between coping appraisal and intention to consume n-3PUFA [17]. In another studies, self-efficacy predicted the gluten consumption [12] as well fruit and vegetable eating behavior [32], based on the PMT among adults with coeliac disease and college students, respectively. In light of these previous findings, the results of this study, which have tested PMT in preventive health contexts, suggest coping appraisal also has a predictive effect on intentions and related behaviors. A demonstrated effect of increased knowledge on coping appraisal could indicate the value of focus on educational interventions.

Although PMT path model showed the significant relationship between self-efficacy and vitamin E and C consumption behavior, the standardized beta coefficients were different. Perhaps the reason for this difference was because of the differences in vitamin E-rich foods with vitamin C-rich foods and also the availability of these workers to food sources containing vitamins E and C. Furthermore, this difference could be related to the dietary habit and the diet of workers in the study area, which had led to a stronger association between self-efficacy and vitamin E consumption behavior than the vitamin C consumption behavior. Socioeconomic characteristics and cultural norms may affect dietary habits and the choice of food and the subsequent individual’s belief to do behavior. This should be further investigated in future studies.

We also found a weak influence of perceived vulnerability on intention. However, this variable and perceived severity did not have a direct influence on behavior of consuming vitamins E and C. Not finding strong associations between perceived vulnerability, perceived severity (threat appraisal), and intention exposes an emerging discord in the literature on why perceived threat showed a limited role in motivating action in the context of vitamin E and C consumption among this population who were exposed to toxic metals. A few studies indicated significant associations in this regard [13, 15]. For example, evaluation of Calder’s study based on PMT and using SEM among university students and employees reported threat appraisal was significantly associated with the consuming of Omega-3 intention [17]. Further research is needed to examine the interactions between knowledge and threat appraisal, as well between threat appraisal and behavioral intention in diverse population and for different behaviors.

In our current study, intention had a significant influence on vitamin E and C consumption behavior; the mediating role of intention between threat and coping appraisal and behavior has been reported [33]. As coping appraisal is more predictive of intention for vitamin consumption behavior than other constructs, interventions could be emphasized among this at risk population. The importance of intention should be considered to create a link between coping appraisal and vitamin consumption behavior. In addition, according to previous PMT research, intention had a significant role and strong predictor on dietary behavioral changes [16, 17]. Therefore, specific attention should be paid to it when designing intervention plans.

While our results are significant, limitations in the study indicate the benefit of expanded sampling. Self-reporting questionnaires introduce the probability of biased outcomes, including recall bias. Furthermore, we did not have access to blood samples. Testing blood samples for vitamin saturation would give a clearer understanding of consumption behaviors. There was no conducting concurrent validity to measure vitamin E and C consumption by FFQ and blood vitamins.

Future studies are warranted to examine PMT constructs and also the role of knowledge at multiple time points. Because of different designs, settings, used theories, and statistical methods that could influence the reliability of results [34], it is suggested that a laboratory experiment measuring blood concentration levels of vitamins E and C be used along with FFQ in order to establish concurrent validity and to increase the accuracy in vitamin use evaluation. In this study, values of vitamins were far from the minimum recommendations to consume vitamins E (15 mg/day) and C (90 mg/day) [35]. Moreover, there is not sufficient support from factory officials to educate workers and check for vitamin deficiency probability in them. Therefore, training high-risk workers to get blood vitamin E and C testing and more education regarding vitamin deficiency levels are necessary. Investigation into what other factors might influence vitamin E and C consumption is warranted. Socioeconomic characteristics and cultural norms beliefs may affect food habits and should be considered.

## Conclusion

To conclude, considering that response efficacy, self-efficacy, and intention showed as strong predictors of vitamin E and C consumption behavior, specific attention should be paid to coping appraisal and intention when designing intervention plans and their strong link to related behavior. Establishing a predicting role of knowledge for PMT constructs in this population should be considered too in intervention programs. Given that the rapid overgrowing urbanization and industrialization have introduced harmful metals, especially heavy metals over the past few decades, growing concern has been focused on effective ways of protection from adverse effects of exposure to these heavy metals. Antioxidant vitamins provide protection against harmful metal-mediated free radical attacks. Deficiency of these vitamins has been shown to deteriorate the toxic effects of metals, and consumption of such nutrients mitigates the toxicity. Therefore, investigation into what factors might influence antioxidant vitamin consumption like E and C is warranted, especially in high-risk groups such as factory workers with high exposure to toxic metals.

## Abbreviations

CFI: Comparative fit index
FCT: Food composition table
FFQ: Food frequency questionnaire
GFI: Goodness of fit index
H: Hypothesis
PMT: Protection motivation theory
RMSEA: Root mean square error of approximation
SEM: Structural equation modeling
TLI: Tucker Lewis index
USDA: United States Department of Agriculture
